# Connexin Communication Compartments and Wound Repair in Epithelial Tissue

**DOI:** 10.3390/ijms19051354

**Published:** 2018-05-03

**Authors:** Marc Chanson, Masakatsu Watanabe, Erin M. O’Shaughnessy, Alice Zoso, Patricia E. Martin

**Affiliations:** 1 Department of Pediatrics and Cell Physiology & Metabolism, Geneva University Hospitals and University of Geneva, 1211 Geneva, Switzerland; marc.chanson@unige.ch (M.C.); alice.zoso@unige.ch (A.Z.); 2 Graduate School of Frontier Biosciences, Osaka University, Osaka 565-0871, Japan; watanabe-m@fbs.osaka-u.ac.jp; 3 Department of Life Sciences, School of Health and Life Sciences, Glasgow Caledonian University, Glasgow G4 0BA, UK; Erin.OShaughnessy@gcu.ac.uk

**Keywords:** epithelial tissue, connexin, pannexin, wound healing, inflammation, zebrafish models

## Abstract

Epithelial tissues line the lumen of tracts and ducts connecting to the external environment. They are critical in forming an interface between the internal and external environment and, following assault from environmental factors and pathogens, they must rapidly repair to maintain cellular homeostasis. These tissue networks, that range from a single cell layer, such as in airway epithelium, to highly stratified and differentiated epithelial surfaces, such as the epidermis, are held together by a junctional nexus of proteins including adherens, tight and gap junctions, often forming unique and localised communication compartments activated for localised tissue repair. This review focuses on the dynamic changes that occur in connexins, the constituent proteins of the intercellular gap junction channel, during wound-healing processes and in localised inflammation, with an emphasis on the lung and skin. Current developments in targeting connexins as corrective therapies to improve wound closure and resolve localised inflammation are also discussed. Finally, we consider the emergence of the zebrafish as a concerted whole-animal model to study, visualise and track the events of wound repair and regeneration in real-time living model systems.

## 1. Introduction

Connexins are a highly conserved group of transmembrane proteins, with 21 subtypes expressed in a human, which form gap junctions with neighbouring cells to enable intercellular communication and metabolite exchange. Connexin hemichannels in the plasma membrane are typically in a default-closed position but can be induced to open under conditions of cell stress to release adenosine triphosphate (ATP) and other small signalling molecules [1,2]. Pannexins, a family of three sister proteins to the connexins, also form membrane channels and are increasingly associated with inflammatory mediated events [3]. Connexins and pannexins share a common topology consisting of four transmembrane domains that span the plasma membrane; two highly conserved extracellular loops, and intracellular C- and N-terminal domains. Six connexin subunits oligomerise to form a connexon that is trafficked to and inserted into the plasma membrane. Connexins laterally accrete to dock with other hemichannels from neighbouring cells to form an intercellular gap junction [1]. Pannexins also oligomerise to form channels and trafficking to the plasma membrane is dependent on interaction with the actin cytoskeleton [4,5]. Both connexins and pannexins play a key role in coordinating processes that mediate the development and maintenance of tissues in multicellular organisms. Distinct connexin and pannexin expression profiles are observed in different tissues with multiple connexins often expressed within the same cell. For example, in the cardiovascular system connexins (Cxs) 37, 40, 43 and 45 are expressed. In epithelial tissue, such as the airway epithelium and stratified epidermis, Cx43 is still the predominant connexin present with Cxs 26, 30 and 31 in particular emerging to play important roles as discussed below. Each connexin forms transmembrane channels that have unique permeability properties in terms of size, ionic permeability and selectivity [6,7]. Thus, the pattern in which these proteins are expressed is very important to cellular function, with each combination of heteromeric channel conferring unique signalling properties. Heterotypic channels are limited in function by connexin compatibility, predicted to prevent cells following different differential pathways to ‘communicate’ [8]. Connexins have a short half-life, resulting in rapid turnover and, together, these properties enable specialised tissue-specific, spatial and temporal communication compartments. It is well established that connexins participate in liver regeneration after injury [9]. However, less is known about the mechanisms of airway epithelium repair in the adult respiratory system. This review will focus on tissue-specific communication compartments during the dynamic events that occur during the regeneration and repair of epithelial tissue networks including the lung airway epithelium and the epidermis. Wound repair in the whole zebrafish model will also be addressed.

## 2. Connexins in Normal and Repairing Airway Epithelium

### 2.1. Connexins in the Airway Epithelium

The airway epithelium is a fundamental component of the innate immune system by protecting the lung against invading pathogens. The concerted action of ciliated and mucin-secreting cells maintain efficient mucociliary clearance, and regulates the production of anti-inflammatory and antimicrobial molecules [10]. Connexin- and pannexin (Panx)-channels provide a complex communication network that maintains lung homeostasis and modulates host defences in both conductive and respiratory (alveoli) airways [11,12]. The upper airway epithelium and submucosal glands express (identified at the mRNA level and/or protein level) about 10 connexins (Cx26, Cx30, Cx30.3, Cx31, Cx31.1, Cx32, Cx37, Cx43, Cx46). Cx32, Cx43 and Cx46 are found in the alveolar epithelium while Cx37, Cx40 and Cx43 are expressed in the alveolar endothelium. Panx1 is ubiquitously expressed, while little information is yet available for Panx2 and Panx3.

In the conducting airways, the communication network plays key roles within the mucosa barrier. For example, cell-to-cell transfer of ions and second messengers is thought to contribute to mucociliary clearance by hydrating the luminal surface and controlling ciliary beat frequency [13,14]. The latter mechanisms are fine-tuned by Panx1-mediated release of ATP which, in an autocrine manner, regulates mucin and water secretion as well as cilia activity [15,16,17]. Gap junctions are also components of the innate immunity defence system by mediating the cell-to-cell spread of pro-inflammatory and pro-apoptotic signals according to the pathogen recognition receptors (PRRs) activated [18,19]. Hence, connexins and Panx1 are deregulated in terms of activity and/or expression in several pathologies, including chronic rhinosinusitis [20] and cystic fibrosis [21,22].

In the respiratory alveoli, the intercellular network participates in the production of surfactant in response to stretch on the epithelial side of the alveoli [23]. On the endothelial side, connexin and Panx1 channels mediate hypoxia-induced vasoconstriction, leukocyte adhesion and transmigration across the endothelial-alveolar wall [24,25,26]. Finally, it has been proposed that alveolar macrophages attached to the alveolar surface may communicate immunosuppressive signals to alveolar epithelial cells, since leukocyte-specific knockdown of Cx43 enhanced endotoxin-induced lung inflammation [27].

**Figure 1:**
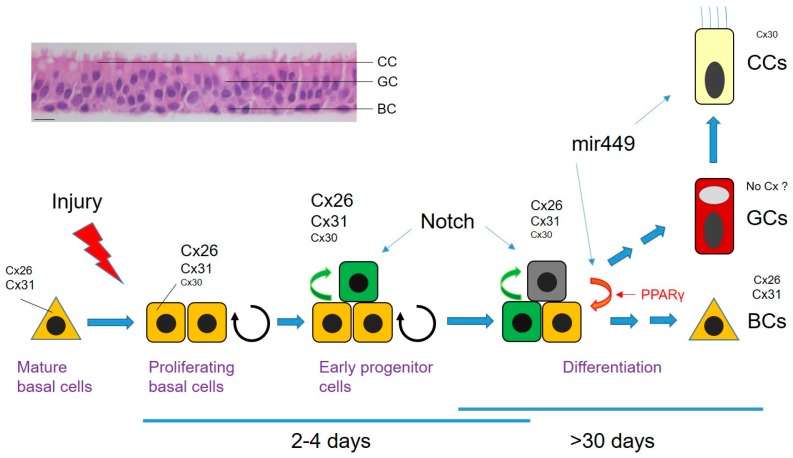
The different phases of airway epithelium regeneration after wounding. The top left images show the histology of the pseudostratified airway epithelium after culturing human airway epithelial cells for 1.5 months on Transwell filter, and the capacity of the epithelium to repair after wounding. BC: basal cell; CC: ciliated cell; GC: goblet cell. The scheme illustrates the steps involved in airway epithelium repair after injury (blue arrows); wound closure is reached within 3–4 days. Orange triangle: CK5-expressing quiescent basal cells; orange square: CK5 and CK14-activated basal cells; light green square: early progenitor cells; dark green square: late progenitor cells. Early differentiation (passage from proliferating cells to early progenitors) is dictated in part by Notch activation (green arrows). Cell division arrest and later differentiation requires increased expression of miR-449. The relative changes in connexin expression (Cx26, Cx30, Cx31) is illustrated for the different stages of the repair process by the size of the fonts. PPARγ signalling also contributes to BC differentiation and decreases connexin expression (red arrow). Bar: 50 µm.

Epithelial repair is a multi-step process initiated by migration of basal cells (BCs) at the leading edge of the wound along with the induction of proliferation to repopulate the injured area [28]. This phase of newly proliferating cells, or blastema, is followed by cell differentiation and patterning (Figure 1). Stem cells and BCs contribute to the regeneration of intact airway epithelium. The BC population is highly heterogeneous comprising morphologically indistinguishable multipotent stem cells and committed precursors [28,29]. The mechanisms that underlie cell-fate specification in repairing airway epithelia are poorly known but several transcription factors, including Yap/Sox and Fox family members as well as Notch family receptors and specific microRNAs, has been reported [28,29,30,31,32,33,34]. For example, Notch signalling is required for the transition of mature BCs to early progenitor cells (EPs), and at later phases for differentiation into goblet/secretory cells (GCs) and ciliated cells (CCs) (Figure 1).

### 2.2. Repair Research in the Adult Airway Epithelium

Re-establishment of the integrity of the airway epithelium is a prerequisite to restoration of the tissue homeostasis and host-defence mechanisms [20]. The tracheobronchial airway epithelium is composed of BCs, GCs and CCs.

### 2.3. Connexins in Wound Repair of the Airway Epithelium

The pattern of connexin expression by the human airway epithelium depends on cell phenotype and the stage of differentiation. Several connexin isoforms are expressed in the undifferentiated airway epithelium of which Cx26 and Cx43 rapidly disappear upon differentiation [35,36]. In the well-polarized epithelium, cell-specific localization of connexin expression is found. Thus, Cx31 connects BCs while Cx30 connects CCs and perhaps BCs to CCs, suggesting distinct compartments of intercellular communication within the airway epithelium. Remnants of Cx26 could be detected, however, in a subset of BCs [36]. At least two populations of BCs can be distinguished by their expression of cytokeratins (CK5 and CK14) whereas CK8 is a marker of EPs and of fully differentiated BCs and CCs. Interestingly, Cx26 expression is strongly induced in CK14-expressing BCs undergoing proliferation in response to wounding [36]. The sustained increase of Cx26 is maintained until wound closure, a point at which the protein progressively returns to its basal level of expression with epithelium differentiation (Figure 1). It is proposed that induction of Cx26-mediated intercellular communication by proliferative signals in activated BCs may represent a means to contain their over-proliferation, which would make them permissive for further differentiation signals. Not only Cx26 but also Cx30 and Cx31 are subjected to modulation during wound repair (Figure 1). At the mRNA level, both Cx30 and Cx31 are upregulated during the proliferation phase to reach a plateau at the time of wound closure. Cx30 detection coincided with the apparition of EPs. The expression of both connexins decreased back to basal levels during the differentiation period (Figure 1). Although an apparent relationship between connexin expression and proliferation can be proposed, the mechanisms involved have not yet been elucidated.

Among the multitude intracellular signalling pathways that control cell-fate specification in lung development and lung-related diseases, a role for the lipid-responsive transcription factor peroxisome proliferator-activated receptor gamma (PPARγ) has been proposed [37,38]. PPARγ is endogenously activated by 15-keto prostaglandin E2 (15kPGE2), which is generated from PGE2 by the activity of hydroxyprostaglandin dehydrogenase (HPGD). In a recent report, Bou Saab and collaborators found that HPGD is involved in the PPARγ-dependent control of the BC population during the repair process [39]. Interestingly, Cx26 expressed in activated BCs was found to be highly sensitive to the differentiating signals mediated by PPARγ [40]. One hypothesis is that PPARγ-induced downregulation of Cx26 may promote in a subpopulation of activated BCs their exit of the cell cycle for further differentiation and/or trigger their return to their original state. Clearly, additional studies are required to understand the regulatory mechanisms and the role fulfilled by the dynamic changes in connexin expression during airway epithelium repair. Gene-silencing approaches using clustered regularly interspaced short palindromic repeats-Cas9 (CRISPR-Cas9) in primary cultures of human airway epithelial cells are anticipated to bring important information on the role of gap junctional intercellular communication in cell fate during airway mucosal regeneration.

## 3. Connexins in the Epidermis

**Figure 2:**
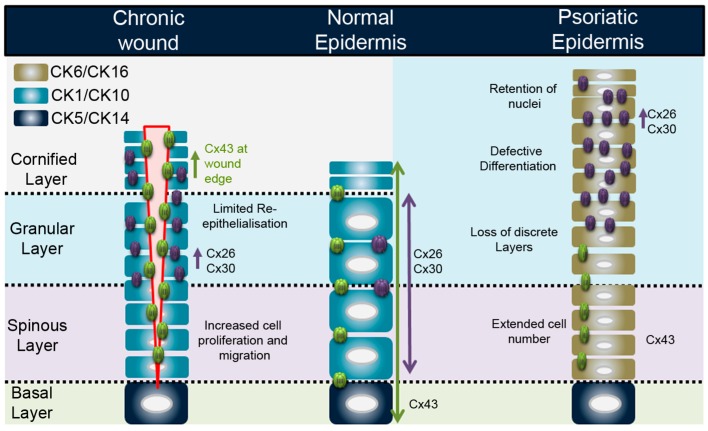
Connexin expression profile in the normal epidermis, at a chronic wound edge, and representative state in a psoriatic epidermis.

The epidermis is a highly specialised, stratified epithelial layer forming a tough barrier to the external environment. It plays a vital protective role from environmental insult and is highly subject to localised trauma and injury resulting in a need for rapid repair of the tissue [41]. The key components of the epidermis are keratinocytes, a specialised subset of cells that attach firmly to the basement membrane of the skin that provides an interface between the vascular dermal layers and the avascular epidermis. A small subset of epidermal stem cells, resident in the hair follicle and interfollicular regions are responsible for the continual renewal of the epidermis [42]. Cells on the basal membrane are, under normal conditions, the only proliferative cells within the epidermis, although only about 15% of cells actively participate in this process, this increases when enhanced proliferation is needed such as in wound healing. Stratification occurs as subsets of the basal layer keratinocytes undergo asymmetrical cell division and enter a differentiation programme resulting in the stratified and cornified epidermis and watertight epidermal barrier (Figure 2). These differentiated layers are characterised by a complex differentiation process typified by changes in the expression profile of keratins, key intermediate filaments of the epidermis. Keratins form pairs with basic and acidic partner complexes; the CK5/CK14 complex is associated with basal keratinocytes and CK1/CK10 expression with cells committed to terminal differentiation pathways [43]. Keratinocyte differentiation results in the transformation of cellular morphology and protein expression, ultimately resulting in a loss of nuclei and the production of keratinohyalin granules in the stratum corneum layer. Keratins interact with a range of proteins including desmosomes, fillagrin, loricrin and keratolinin to give a highly ordered and structured epidermis and the stratum corneum its flattened shape (Figure 2). There is a balance between the renewal and desquamation of the epidermis, a process that takes about 28 days in a man with a normal epidermal profile of about 10 cell layers [43].

Up to 10 different connexin isoforms are expressed with the profile characterised by the differentiation status (Figure 2). Cx43 is the predominant connexin in the basal layers, associated with proliferation, while Cx26 and Cx30 are associated with upper differentiated cell layers. Cx31 tends to be expressed in similar layers to Cx43 while Cx31.1 is only found in the upper differentiated layers and linked with apoptosis and cell shedding [44,45]. Due to the avascular nature of the epidermis, it is generally accepted that gap junctional intercellular communication is central to ensuring correct signalling between the inner and outer layers. Specialised communication compartments have been suggested due to the predicted nature of the α and β connexin interactions and selective permeability properties [6,46]. Recent evidence suggests that Cx43 (a member of the α connexin subgroup) and Cx31 (a member of the β connexin subgroup) can form heteromeric channels, indicating that such interactions could act as a bridging link between epidermal areas where the incompatible Cx43 and β-connexins Cx26 and Cx30 are the predominant connexins expressed [47,48]. In the human epidermis, the two main connexins are Cx26 and Cx43. Cx26 expression is also found in hair follicles and eccrine sweat glands, whereas Cx43 is located in the interfollicular epidermis [44,49,50]. The importance of connexins in the epidermis is further highlighted by the plethora of mutations that are associated with both inflammatory and non-inflammatory hyperproliferative epidermal disorders (recently reviewed by [51,52]). Pannexins 1 and 3 are also expressed in keratinocytes where knockout mouse models revealed an important role for Panx1 in epidermal formation [53,54,55].

### 3.1. Connexins in Epidermal Wound Healing

Skin integrity is dependent on interactions between keratinocytes and the extracellular matrix (ECM), and these are the first cell type to sense injury. The introduction of a wound to the epidermis triggers an acute inflammatory response, keratinocyte migration and proliferation, and induces changes in the cytoskeleton and keratinocyte adhesion [56,57]. In the context of connexins, a key event in normal wound healing is the downregulation of Cx43 at the wound edge within 6 h of injury [58]. This is associated with the activation of migration of keratinocytes into the gap. There is also evidence of specialised spatial communication compartments behind the wound edge where Cx43 is subject to phosphorylation, particularly at P-ser368 on the carboxyl terminal domain [59,60]. The localised switch in phosphorylation probably changes the functional signalling parameters of Cx43 channels, further enhancing migration of wound edge keratinocytes. This may include the induction of signalling pathways, including Transforming Growth Factor-β TGF-β and ECM deposition, all of which are key events in wound closure [61,62,63]. Upon re-stratification, levels of Cx43 are re-established [45,58,64,65]. In other studies, Panx1 was also shown to be elevated at the wound edge during the early stages of wound healing and overall rates of wound closure were reduced in Panx1 Knockout (KO) mice models [66].

### 3.2. Connexins and Inflammation in the Epidermis: Chronic Wounds and Psoriasis

Dysregulation of connexin expression in the epidermis is associated with a variety of conditions, re-enforcing the importance of these proteins in maintaining epidermal integrity. This is most evident for Cx43 and Cx26 where alteration in the fine balance of control of expression is associated with pathological conditions including chronic non-healing wounds, psoriasis and a range of connexin-channelopathies linked with mutations in β-connexins causing skin disease [8,51].

Chronic non-healing wounds are maintained in a high inflammatory state and are subject to infection. These are increasingly associated with situations such as diabetic ulcers and lower limb disease, but also with conditions such as pressure sores, prevalent in ageing societies, increased obesity and diabetes rates [57,67]. Such events place enormous burdens on healthcare resources and management. Although many novel therapies are under development, including hyperbaric oxygen therapy, growth factors and stem-cell implantation, current treatments only manage the condition by frequent debridement therapy and pressure off-loading [68]. In terms of connexin expression, Cx43 is significantly upregulated at the wound edge of chronic non-healing wounds in both diabetic and non-diabetic patients [58,69,70]. Such changes in expression significantly alter the dynamic and subtle crosstalk between cells with the local area; it is proposed that it plays a vital role in the sustainment of the non-healing wound state. Thus, Cx43 in particular has become a prime therapeutic target to improve wound healing with both antisense Cx43 and Cx43 peptidomimetic strategies exhibiting exciting opportunities [71] (see Section 5). In chronic non-healing wound margins, reports have also determined that Cx26/Cx30 expression is significantly enhanced and associated with the hyper-proliferative and inflammatory skin phenotype [70].

Psoriasis is another chronic inflammatory skin condition affecting 2–3% of the population. The classic phenotype of psoriasis can be likened to that of a chronic wound with its persistent inflammation and changes to the healing profile of the epidermis [72]. Persistent plaques that vary in size and depth form the prototypic form of psoriasis, Psoriasis vulgaris, often described as the Koebner phenomenon with well defined, silvery-white scaly skin areas of skin lesions. The leading hypothesis on disease initiation in psoriasis is T-cell driven via the immune system (inside-out hypothesis); however, recent data suggest that the environment may play a role (outside-in), including shifts in the skin microbiome [73,74], epidemiological factors such as geographical location [75], and epidermal barrier disruption [76,77]. Genetic factors also play a role, with several loci identified as psoriasis susceptibility risk factors. The most prominent is *PSOR1*, a Major Histocompatibility Complex (MHC) Class 1 region on the chromosome 6p21 [78], and a number of reports suggest that polymorphisms on *GJB2* represent suitable markers of susceptibility [79,80,81,82].

Enhanced Cx26 and Cx30 protein expression is highly evident in psoriatic plaques and a transcriptome analysis revealed *GJB2*, to be among the top 100 genes upregulated in psoriasis, with its expression levels increased up to 18-fold in psoriatic lesions compared to normal tissue [50,51,83,84,85]. Epidermal hyperplasia in the psoriatic epidermis drives an accelerated growth and altered differentiation of the keratinocytes that results in a loss of the discrete epidermal layers. The highly proliferative basal layer leads to an extended cell number in the spinous layer and a merging of the granular and cornified layers. Nuclei are retained in the outer layers and there is an overall defective terminal differentiation, which drives a change in keratin expression from the normal CK1 and CK10 to CK6 and CK16, characteristic markers for psoriasis [72,86]. In support of a role for the over-expression of Cx26 in driving some of these events, transgenic mice studies over-expressing Cx26 in suprabasal keratinocytes exhibited pathological features similar to those seen in psoriasis, with the suggestion that enhanced Cx26 hemichannel activity plays a central role [87]. Several further lines of evidence suggest that susceptibility to psoriasis is linked to the epidermal differentiation complex, including downregulation of E-cadherin and Cx43 and enhanced expression of Claudin13, a key component of epidermal tight junctions [76,88,89,90,91,92]. 

## 4. Disruption of Cx43:Cx26 Balance in Epithelial Tissue: Connexins and the Environment

Maintaining homeostasis with the microbiome is a critical feature of all epithelial tissues [93,94]. Dysbiosis of the skin microflora has been associated with many dermatological conditions including but not limited to psoriasis and chronic non-healing diabetic wounds. Bacterial colonisation of psoriatic skin shows a shift from commensal organisms, such as *Staphylococcus epidermidis,* to more opportunistic pathogens such as *S. aureus* [73,95] and shifts in skin flora of diabetic patients and non-healing wounds are highly evident. The microbiome of the respiratory system is less well characterized but recent reports showed its alteration in diseases such as Cystic Fibrosis CF. The lungs of CF patients are normal *in utero* and in the newborn period, and are usually colonized by a variety of opportunistic bacterial, viral and fungal pathogens in an age-dependent sequence [94]. The most frequently found organism during colonization of CF airways is *S. aureus*, followed later by *Pseudomonas aeruginosa*, which remains the critical determinant of pulmonary pathology in the late stages of the disease.

Accumulating evidence suggests that exposure of epithelial cells to components of opportunistic pathogens, such as *S. aureus*, *P. aeruginosa* and *Shigella flexneri*, can alter connexin hemichannel activity and connexin expression levels [77,96,97]. In the context of the epidermis, we previously determined that exposure of keratinocytes to peptidoglycan isolated from the cell wall of the opportunistic skin pathogen *S. aureus* induces Cx26 expression with associated links to inflammation, while that isolated from the commensal *S. epidermidis* is without effect [77]. In parallel studies in other tissue networks, bacterial colonisation has been shown to trigger connexin signalling including the glial, intestine and lung epithelia [98]. Hence, controlling the level of Cx expression and/or function in these specialised tissue niches and regulating the balance of Cx43 and Cx26 is a critical focus for maintaining epithelial integrity. Further studies suggest that acute exposure to pro-inflammatory mediators, such as peptidoglycan or lipopolysaccharide, is sufficient to trigger connexin hemichannel activity and release of secondary messengers such as ATP, nicotinamide adenine dinucleotide (NAD^+^), glutamate and prostaglandins [99,100]. Following the release of ATP, the activation of purinergic signalling cascades occurs that plays a central role in differentiation and proliferation of the epidermis as well as regulation of innate immune responses [101]. In conditions such as psoriasis or chronic non-healing wounds where connexin expression levels are excessive, the localised release of ATP could be in part responsible for exacerbated pro-inflammatory responses, altering intracellular calcium dynamics leading to changes in the terminal differentiation programme and a hyperproliferative state [102,103,104,105].

Similarly, the release of extracellular ATP is thought to amplify the inflammatory response evoked by *P. aeruginosa*-dependent infection of the airway epithelium. Interestingly, both connexins and Panx1 were found to be involved in ATP release at the airway mucosa [106,107]. However, protective or deleterious outcomes of ATP on inflammation may depend on the activated purinergic receptors. For example, the release of ATP from airway epithelial cell via Panx1 channel and subsequent activation of P2Y11 contributes to the resolution of inflammation and triggers wound repair [21]. 

## 5. Connexins as Therapeutic Targets in Epithelial Tissues

De-regulation of connexin expression is thus a key event in epithelial pathology. To enable the dissection of the molecular mechanisms underpinning these events, a range of studies utilising knockout and antisense technologies and connexin peptidomimetics have provided extensive information and revealed exiting therapeutic strategies [108].

In targeted epidermal ablation of Cx43 in mouse models, wound-healing rates were significantly faster than in normal mice. The reduction of Cx43 showed that the keratinocyte layer was much thinner with a wound closure rate that more than doubled, reaching or surpassing the rates of the untreated controls [65,109]. Early studies by Becker and Green developed a topical application of an antisense oligonucleotide targeted to Cx43, reducing Cx43 expression and improving wound closure rates in both “normal” and diabetic rat wound-healing models. Day one observations of epidermal regrowth determined that, after injury, diabetic skin showed no re-growth compared to the controls, but when treated with the Cx43 antisense oligonucleotide the regrowth matched that of the controls [110,111,112,113]. This strategy was taken forward to clinical trials providing powerful evidence for the development of Cx-therapeutic strategies. Significantly, these studies provided evidence that reduction of Cx43 reduced the inflammatory status, altered ECM deposition and reduced scarring [114,115,116].

Although an antisense approach is applicable, there is much controversy over channel versus non-channel function. A peptide targeted to the carboxyl terminal domain of Cx43, thereby interfering with ZO-1 interactions, was identified as a potent regulator of Cx43 channel function. Application of this peptide, ACT-1, to wounds in animal models determined that it enhanced wound-closure rates and reduced inflammation, ECM deposition and scarring, without altering *Cx43* gene expression. It is the first in its class of connexin peptidomimetics to be successfully applied in clinical trials showing profound improvement in healing rates of venous leg ulcers [117,118,119,120]. Further mimetic peptides that mimic the extracellular loops of connexins (Gap26 and Gap27) have been reported to enhance wound-closure rates in in vitro 2D and 3D human and mouse epidermal models. Gap27 blocks hemichannel and gap junctional communication without influence on *Cx43* gene expression in keratinocytes [64,121,122,123]. It enhances keratinocyte migration rates, without altering cell proliferation supporting concepts that hemichannel activity is involved in keratinocyte galvanotaixis [61,124].

Less is known regarding the targeting of connexins and Panx1 in the airway epithelium. In a murine bleomycin model of acute lung injury, intravenous administration of a Panx1 mimetic peptide reduced the presence of leukocytes in the bronchoalveolar lavage fluid [125]. Similarly, neutrophils’ recruitment to the airspace in response to lung lipopolysaccharide (LPS) administration was reduced by intratracheal instillation of Gap26 during the course of inflammation [26]. Whether this inhibition occurs at the epithelium, endothelium or leukocyte level is not clear. Finally, the effects of peptidomimetics on airway epithelium repair have not yet been reported.

## 6. Zebrafish Connexins in Wound Repair and Regeneration

Zebrafish are an emergent model organism used to study development and regeneration because of their unique advantages, including their small body length (2–3 cm), easy breeding, large number of fertilised eggs (over 100) obtained from a single pair of zebrafish at one time, and short generation time (3 months). The embryos usually hatch and start swimming at 3 days after fertilisation and begin feeding about 5 days after fertilisation, indicating that the development of the nervous system, motor function, and digestive organs is almost complete in this short period. Furthermore, zebrafish have a transparent embryo body, facilitating the observation of development by live imaging. In addition, genome information and genetic-modification methods, including Tol2-mediated gene transfer and CRISPR gene knockout, have been utilised in zebrafish [126,127]. These tools have enabled confirmation of these phenomena, as shown in other organisms, and have accelerated biological research.

### 6.1. Wound-Repair Research in Zebrafish

In addition to skin and bone, which are common targets in wound-repair and regeneration studies, nerves, heart, retina, tendon and muscles are actively evaluated in studies utilising the high regeneration capacity of zebrafish [128,129]. Furthermore, by exploiting the transparency characteristics of zebrafish embryos, live cell imaging of wound repair has been performed. In a recent report, for example, the H_2_O_2_ concentration gradient occurring in skin wounds was successfully visualised, and macrophages accumulated at the wound point on the skin in an H_2_O_2_ concentration-dependent manner [130].

### 6.2. Zebrafish Connexins

With regard to gap junction genes, 38 connexin and four pannexin genes are predicted in the zebrafish genome. The higher number of connexin genes in zebrafish compared with that in humans is due to gene duplication events, which occurred in the teleost lineage during fish evolution [131]. Because expression analysis and functional analysis have not been performed for all of these genes, it is unclear how many genes are actually functional. Cx43 is the most abundantly expressed connexin in many organs and is being actively studied. Zebrafish (Zf) Cx43 and human Cx43 have a very high identity of 80% at the amino acid level, and functional conservation between ZfCx43 and mammalian Cx43 was analysed and confirmed [132,133]. As a connexin specific to the teleost lineage, ZfCx39.4 is not present in the genome of amphibians or higher organisms. Interestingly, ZfCx39.4 is involved in skin pattern formation in zebrafish, together with ZfCx41.8, which is an orthologue of mammalian Cx40 [134,135].

### 6.3. Zebrafish Connexins in the Heart

Unlike epithelial tissues discussed above, mammalian hearts do not regenerate because cell division ceases after birth; however, recent studies have confirmed that partial cell division ability is retained [136]. On the other hand, amphibians and fish have higher regeneration ability than mammals, and wounded hearts regenerate well in these organisms because differentiated cardiomyocytes can undergo dedifferentiation and proliferation throughout the organisms’ lifetime [137].

The expression of ZfCx36.7 (an orthologue of human Cx31.9), ZfCx43 (an orthologue of Cx43), ZfCx41.8 (an orthologue of Cx40), ZfCx45.6 (an orthologue of Cx45), and ZfCx48.5 (an orthologue of Cx 46) have been reported in zebrafish hearts [138,139,140,141]. Among these proteins, mutations in ZfCx36.7 and ZfCx48.5 have been isolated from mutant zebrafish with heart defects, indicating that these connexins are important for maintaining heart function. In contrast, no defects have been detected in the hearts of ZfCx41.8 and ZfCx45.6 mutants, although knockout of *Cx40* in mammals causes severe defects in heart function. With regard to Cx43 in zebrafish, expression was detected in normal and regenerating hearts; however, the role of Cx43 in regeneration is still unclear [142,143].

### 6.4. Zebrafish Connexins in the Fin

The fin of the zebrafish is composed of segmented hemirays of bone matrix that surround mesenchymal cells. The amputation of the fin follows subsequent steps, wound healing, blastema formation and regenerative outgrowth. During this process, osteoblasts function both in fin bone elongation and joint formation, and the fin returns to its original form in about 10–14 days [137]. 

As a connexin involved in the development and regeneration of the fin, Cx43 has been isolated from a zebrafish mutant, *short-of-fin* (*sof*), which exhibits shorter fin segment length compared with that of wild-type fish [133]. Four alleles of the *sof* mutant have been isolated. One is a mutant having low expression of *Cx43* mRNA but no amino acid substitution in the protein, and the other three have amino acid substitutions in Cx43. Electrophysiological analysis has been performed for each of the three mutants, indicating that the electrical properties of gap junctions are well linked to caudal fin length in adult fish [144]. 

In a model of Cx43 function in fin regeneration, it was proposed that Cx43 controls the switch for elongation of fin length or for initiation of joint formation [145]. Cx43 is expressed in regenerating fin mesenchyme and cells surrounding the bone joint, and expression of *cx43* was correlated with cell proliferation during fin regeneration. In the *cx43* mutant, decreased expression levels were detected and the cell proliferation rate was also decreased, resulting in shortening of fin length [146]. Loss of Cx43 function also causes premature joint formation, leading to shorter fin ray segments [145,146,147]. Several genes acting downstream of *cx43* were identified. The *evx1* gene is required for joint formation in the zebrafish fin [148]. Indeed, *evx1* mutant fish do not form joints, although normal development and regeneration in fin formation are observed [149]. Recent studies determined that Cx43 can regulate *evx1* expression [150]. Independent of *evx1* function, Cx43 controls the switching of fin growth and joint formation through Semapholin3d (Sema3d) function [148,151]. This process involves Cx43 activating Sema3d, which suppresses joint formation through the receptor, PlexinA3. At the same time, Sema3d also induces cell proliferation by suppressing the Neuropilin2a receptor, which has negative effects on cell proliferation. In contrast, when Cx43 function is suppressed, cell proliferation is also suppressed because Neurpilin2a is activated, and joint formation is activated by negative regulation of PlexinA3. Furthermore, with regard to cell proliferation, Hela1n1a protein is involved in the stabilisation of Sema3d function [152,153].

As a zebrafish-specific phenomenon, Cx43 function may be influenced by ZfCx40.8, which is a paralogue of Cx43. As described above, ZfCx43 has 80% identity at the amino acid level with human Cx43, whereas ZfCx40.8 has only 63% identity with human Cx43. This lower identity is due to the presence of a low homology region in the C-terminal domain of ZfCx40.8, suggesting that factors causing functional diversification of ZfCx40.8 exist in this region. Moreover, ZfCx40.8 is thought to be localised to and function in the cell membrane at the time of ontogeny, but remains in the Golgi at the time of fin regeneration. In previous in vitro experiments, the C-terminal domain of ZfCx40.8, which shows low homology to Cx43, was found to control the difference in membrane localisation of ZfCx40.8 between generation and regeneration of fins. Differences in membrane localisation of ZfCx40.8 may be involved in controlling the promoting function of ZfCx43 for cell division or differentiation [154,155].

## 7. Concluding Remarks

Connexin expression profiles within epithelial tissue networks are dynamically regulated following wounding and assault from external pathogens. These events are localised and result in changes in spatial, tissue-specific, communication compartments that allow tissues to respond to repair processes. We have reviewed recent understanding of the connexin communication compartments within the airway epithelia and epidermal networks. Another stratified epithelium surface, continually exposed to the external environment and renewed via a subset of specialised stem cells, is the cornea [42]. Corneal wound healing is a complex process that has many similarities to epithelial, stromal and endothelial cells. Wound healing of the corneal epithelium involves cell migration, cellular proliferation, adhesion, differentiation and cell-layer stratification much like is observed in the epidermis, and injury of the cornea again results in dysregulation of Cx43 expression. In addition to the studies in the skin, accumulating evidence highlights connexins as advanced therapeutic targets for corneal wound repair with both antisense Cx43 and peptidomimetic strategies proving advantageous, accelerating wound healing and reducing inflammation [156,157,158]. Furthermore, Gap27 was recently reported to promote migration during corneal repair, but had a limited effect on deeper vascular tissue and inflammation, again illustrating the importance of these complementary strategies in determining channel versus non-channel functions in specific subcellular compartments [159].

In conclusion, the molecular mechanisms that are driven by altered signalling caused by shifts in connexin expression profiles are now beginning to be resolved. The use of 3D ex vivo model systems, CRISPR-Cas9 technologies, and the emergence of the zebrafish and its genetic modification capabilities, together with the panel of tools to modify connexin function, provide pivotal tools for future studies. Although transfection of primary epithelial cells is challenging, lentiviral vectors can be used to introduce large transgenes into dividing or quiescent cells; thus, knockdown of target genes by delivery of CRIPR-Cas9 has already been achieved [160].

## Abbreviations

15kPGE2	15-keto prostaglandin E2
ATP	adenosine triphosphate
BC	basal cell
CC	ciliated cell
CF	cystic fibrosis
CK	cytokeratin
CRISPR-cas9	clustered regularly interspaced short palindromic repeats-Cas9
Cx	connexin
ECM	extracellular matrix
EP	early progenitor cell
GC	goblet /secretory cell
HPGD	hydroxyprostaglandin dehydrogenase
KO	knockout
LPS	lipopolysaccharide
Panx	pannexin
PGN	peptidoglycan
PPARγ	peroxisome proliferator-activated receptor gamma
TGF-β	transforming growth factor β
Zf	zebrafish
